# Hyperuricemic PRP in Tendon Cells

**DOI:** 10.1155/2014/926481

**Published:** 2014-09-08

**Authors:** I. Andia, E. Rubio-Azpeitia, N. Maffulli

**Affiliations:** ^1^Regenerative Medicine Laboratory, BioCruces, Cruces University Hospital, 48903 Barakaldo, Spain; ^2^Department of Musculoskeletal Disorders, School of Medicine and Surgery, University of Salerno, 89100 Salerno, Italy; ^3^Queen Mary University of London, Barts and the London School of Medicine and Dentistry Centre for Sports and Exercise Medicine, Mile End Hospital, 275 Bancroft Road, London E1 4DG, UK

## Abstract

Platelet-rich plasma (PRP) is injected within tendons to stimulate healing. Metabolic alterations such as the metabolic syndrome, diabetes, or hyperuricemia could hinder the therapeutic effect of PRP. We hypothesise that tendon cells sense high levels of uric acid and this could modify their response to PRP. Tendon cells were treated with allogeneic PRPs for 96 hours. Hyperuricemic PRP did not hinder the proliferative actions of PRP. The gene expression pattern of inflammatory molecules in response to PRP showed absence of IL-1b and COX1 and modest expression of IL6, IL8, COX2, and TGF-b1. IL8 and IL6 proteins were secreted by tendon cells treated with PRP. The synthesis of IL6 and IL8 proteins induced by PRP is decreased significantly in the presence of hyperuricemia (*P* = 0.017 and* P* = 0.012, resp.). Concerning extracellular matrix, PRP-treated tendon cells displayed high type-1 collagen, moderate type-3 collagen, decorin, and hyaluronan synthase-2 expression and modest expression of scleraxis. Hyperuricemia modified the expression pattern of extracellular matrix proteins, upregulating COL1 (*P* = 0.036) and COMP (*P* = 0.012) and downregulating HAS2 (*P* = 0.012). Positive correlations between TGF-b1 and type-1 collagen (*R* = 0.905,* P* = 0.002) and aggrecan (*R* = 0.833,* P* = 0.010) and negative correlations between TGF-b1 and IL6 synthesis (*R* = −0.857,* P* = 0.007) and COX2 (*R* = −0.810,* P* = 0.015) were found.

## 1. Introduction

The use of platelet-rich plasma (PRP) to treat tendon pathology has widely expanded in the last five years [1]. PRP is injected within tendons aiming at healing, reducing pain, and improving tendon function [2]. A recent meta-analysis has shown a significant reduction of pain at three years, six years, and one year after PRP treatment in different tendons [3].

PRP injection is an autologous treatment derived from the patient's own blood; thus metabolic alterations such as the metabolic syndrome, diabetes, or hyperuricemia could hinder the therapeutic effect of autologous PRP in these patients. Despite a higher risk of suffering tendinopathy [4], patients with metabolic diseases are often excluded from the clinical trials testing PRP efficacy [5, 6]. However, whether metabolic disorders should be an exclusion criterion from PRP therapies is open to question.

Hyperuricemia is a relatively common metabolic disease with a prevalence of more than 10% in certain populations [7]. When uric acid, the end product of purines metabolism, rises above 6.8 mg/dL in peripheral blood, urate sediments can form within tissues. Actually, hyperuricemic patients have supersaturated levels of uric acid in their body fluids, and a minority develop gout when uric acid condensates and forms monosodium urate crystals (MSU). The latter can deposit in skin, tendons, and synovium [8] stimulating acute inflammatory flares.

Additionally, extracellular uric acid concentration rises locally upon cell death. In fact, cells normally contain very high levels of uric acid intracellularly [9] and produce even more upon death, signalling danger. Cell death is a pathological event in tendinopathy, affecting not only the area where the tendinopathy is located but also the adjacent tendon tissue [10]. Thus, the response of tendon cells to hyperuricemia may help to understand some aspects of tendinopathy. Recent data show that high serum uric acid is associated with hypertension, kidney disease, cardiovascular disease, and diabetes [11]. However, the association of hyperuricemia with tendinopathy is still ambiguous.

Previous studies have described the pathophysiological role of MSU crystals in alerting the immune system to danger, and the possibility of suffering harm is sensed by monocytes/macrophages that drive an inflammatory reaction by releasing active IL-1b [12]. Whether other cell types, molecular sensors, and synergic mediators participate in the inflammatory response is being explored. For example, serum amyloid A (SAA) protein primed synovial fibroblasts to produce active IL-1b and IL-1a when exposed to MSU crystals [13]. SAA is a proinflammatory protein present in PRP. In addition to SAA and uric acid, PRP also contains other proinflammatory stimuli of self-origin that transmit danger signals including hyaluronan fragments, ATP, DNA, RNA, and HMGB1 [14]. The levels of uric acid are the same in PRP as in serum (3.4–7.2 mg/dL in men and 2.4–6.1 mg/dL in women), since uric acid is not a platelet-derived product but a result of hepatic metabolism.

We raised the hypothesis that tendon cells sense elevated levels of uric acid and this could modify their response to PRP. We explored whether hyperuricemic PRP induces inflammatory, phenotypic, or metabolic changes in tendon cells. To test this hypothesis, we examined in parallel the response of tendon cells to PRP and hyperuricemic PRP exposure by assessing the expression of specific tendon tissue molecules, including type 1 collagen (COL1A1), scleraxis (SCX), decorin (DCN), tenomodulin (TNMD), cartilage oligomeric protein (COMP), and aggrecan (ACAN). In addition, the expression of molecules that characterize early tissue repair such as type 3 collagen (COL3A1) and hyaluronan synthase 2 (HAS2) was determined as well as the expression of inflammatory modulators IL-1b, IL-8, COX-1, and COX2. Finally, we have explored whether hyperuricemic PRP may influence the synthesis of pleiotropic cytokines such as TGF-beta1 and IL-6, which might have a role in enhancing collagen synthesis [15, 16].

## 2. Materials and Methods

### 2.1. Demographic Characteristics of PRP and Cell Donors

We have used primary tendon cells (up to passage 3) isolated from three young male healthy donors (T1, T2, and T3) of similar age (27 ± 1.4 years), in order to minimize heterogeneity of cells among donors. On the other hand, we have used PRP from six donors, three male and three female donors with different age (median age = 41.5 years, range = 26–62) and hormonal status. The mean uric acid and cholesterol levels were 4.4 ± 0.8 mg/dL and 223.8 ± 44.2 mg/dL. The PRPs contained 2.29 ± 0.50 fold peripheral blood platelet count, and mean platelet volume was 7.65 ± 1.01 ftL. Leukocytes were not detected.

### 2.2. Isolation and Culture of Tendon Cells

Human tendon samples were obtained, under anonymous conditions, from three young healthy patients during anterior cruciate ligament reconstruction surgery with semitendinosus tendon, after informed consent and local ethic committee approval. Tendon fragments, which otherwise would have been discarded, were minced and incubated with active 0.3% Collagenase II (Gibco, Life Technologies) at 37°C for 40 min. The cell suspension was centrifuged, resuspended in DMEM F-12 (Gibco, Life Technologies) supplemented with 5% Penicillin/Streptomycin solution (5,000 U/mL Pen. 5,000 *μ*g/mL Strep. Gibco, Life Technologies), filtered, and seeded in a 6-well plate. Cells were allowed to grow until subconfluence and then were trypsinized (TryPLE select 1x, Gibco, Life Technologies) and passaged to a T75 flask at a density of 4000 cells/cm^2^. All the experiments were performed in cells at passages between two and three.

### 2.3. Preparation of PRP and Hyperuricemic PRP

Pure PRP, the same formulation we use in clinical applications, was obtained by single spin method as previously described [17]. Leukocyte and platelet counts were assessed in peripheral blood and PRP using a Beckman Coulter.

Platelet activation and lysis were performed by three freeze thaw cycles, then filtered through 0.22 *μ*m filters, and stored frozen at −80°C. In cell culture, heparin was added at 2 U/mL to PRP lysates. A supersaturated solution of uric acid (Sigma Cat. no. U2625) was prepared with 1 mg uric acid in 1 mL of DMEM F12 (100 mg/dL). The final concentration of uric acid in hyperuricemic PRP cultures was ≈20 mg/dL, that is, the upper concentration in most kits designed for hyperuricemic patients to monitor their uric acid levels in blood.

### 2.4. Cell Proliferation Assays

Cells were harvested from T75 flasks after trypsinization (TryPLE select, Gibco, Life Technologies) and seeded in 96-well plates (Corning) at a density of 4000 cells/cm^2^ and starved overnight before treatments were performed. Cells were treated with PRP lysate or hyperuricemic PRP lysate from the six donors; in parallel, as a reference, cells were cultured with 10% FBS. Cell proliferation was measured at 0, 24, 48, 72, and 96 h with the XTT method.

Population doubling time was used to determine proliferation rate as a total culture time divided by the number of generations calculated as Log 2^*N*_*c*_/*N*_0_^, where *N*_*c*_ is the population at confluence and *N*_0_ is the seeded cells.

### 2.5. RNA Extraction and Real-Time RT-PCR

Total RNA was extracted from tenocytes at passages 2-3, after 4 days of PRP treatment using High Pure RNA Isolation Kit (Roche), following manufacturer instructions. RNA concentrations were measured with the NanoDrop 2000 (Thermo Scientific, Waltham, MA, USA).

1 *μ*g RNA was reverse-transcribed to cDNA using random hexamers in 20 *μ*L, (SuperScript III First-Strand Synthesis System, Invitrogen, Life Technologies). For the real-time PCR, cDNA from each sample was diluted 5-fold and 2 *μ*L of cDNA (20 ng) was mixed with Power SYBR Green PCR Master Mix (Applied Biosystems, Life Technologies) and 5 pmoles of primers to a final volume of 20 *μ*L. Real-time PCR reactions were performed on the ABI-7900 (Applied Biosystems, Life Technologies, Carlsbad, CA, USA). The PCR reactions were performed in triplicate for each sample.

We assessed gene expression for tendon tissue markers scleraxis (SCX), decorin (DCN), tenomodulin (TNMD), matrix proteins including COMP, COL1A1, and COL3A1, and the enzyme for HA synthesis, HAS2. In addition, the expression of cartilage markers COL2A1, aggrecan, and SOX9 was assessed. Likewise inflammatory modulators such as IL-1b, COX1, COX2, and pleiotropic cytokines including IL-6, IL-8, and TGF-beta1 were assessed. Amplification reactions were performed for GAPDH and TBP as reference genes. Primers and annealing temperatures are shown in Table 1 [18, 19]. Standard curves were generated for every gene; the slope of the curves was always between 3.2 and 3.7. Relative expression levels of tendon cells treated with PRP were normalized using glyceraldehyde 3-phosphate dehydrogenase (GAPDH) and calculated by means of the 2^−ΔCt^ method (Ct, cycle threshold).

To assess the effect of hyperuricemia, relative expression levels were normalized to the average of GAPDH and TBP, and gene expression data were calculated as fold versus control using the 2^−ΔΔCt^ (hyperuricemic PRP versus PRP).

### 2.6. Assessment of IL-6 (CXCL6) and IL-8 (CXCL8) in the Conditioned Media

IL-6 and IL-8 were measured in cell culture supernatants using EASIA kits (Invitrogen, Life Technologies). The procedures were performed according to the manufacturer's instructions. Briefly, the reaction was detected by peroxidase-conjugated streptavidin followed by a substrate mixture that contained hydrogen peroxidase as a substrate and ABTS as chromogen. The absorbance was measured in a microplate ELISA reader (PolarStar Omega, BMG Labtech, Offenburg, Germany) at 450 nm, and the concentration was calculated using standard curves. The contribution of 10% PRP was subtracted in order to obtain the cytokine amount produced by cells.

### 2.7. Statistical Analysis

The experiments were performed in triplicate for each of the six PRP donors per three tendon donors. The effects of PRP on proliferation are shown as means ± standard deviation (SD). The effect of PRP on tendon cells expression is shown as median and 25–75 percentiles. Spearman coefficient was used to describe correlations. The effect of hyperuricemic PRP was expressed as the mRNA ratio of hyperuricemic PRP versus PRP expression. *P* values were determined using Student's *t*-test or Wilcoxon test for nonparametric matched values. A *P* value of less than 0.05 was considered to be significant. Data were analyzed using SPSS 18 (SPSS, Chicago, IL, USA).

## 3. Results

### 3.1. Hyperuricemic PRP Does Not Interfere with PRP Induced Proliferation

After 96 hours in culture, PRP significantly enhanced tendon cell proliferation when compared to FBS (*P* = 0.008). The doubling time of the tenocyte population was 40.365 (SD = 2.34) hours in the presence of 10% PRP and 44.72 (SD = 1.14) hours when cultured with 10% FBS.

**Figure 1:**
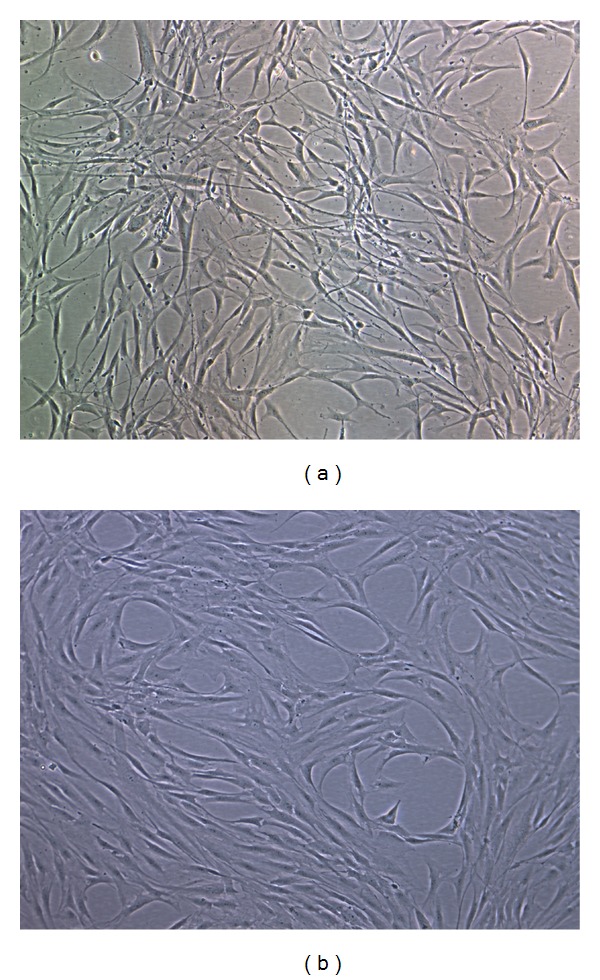
Representative images of tendon cells (passage 2) (a) treated with PRP for 96 hours and (b) treated with hyperuricemic PRP for 96 hours, magnification 20x.

Hyperuricemia did not affect cell proliferation; the tenocyte numbers increased by 548% and 545.5%, respectively (*t* = 0.59; *P* = 0.954). The population doubling time of the cells cultured in PRP was 40.40 hours (SD = 6.105); the doubling time of the population of cells cultured in hyperuricemic PRP was 39.97 hours (SD = 5.265) (Figure 1).

### 3.2. Response of Tendon Cells to PRP Treatment: Gene Expression Pattern of Inflammation and Extracellular Matrix

After 96 hours of treatment with PRP, the tendon cells showed modest expression of IL-6, IL-8, and COX2 (Table 2). Also, the tendon cells treated with PRP showed high expression of type 1 collagen and moderate expression of type 3 collagen and HAS2. The expression of scleraxis and COMP was low in T1 and T2 and moderate in T3. These cells also showed a moderately low expression of TGF-b1. Instead, tendon cells treated with PRP did not express TNMD, COL2A1, SOX9, IL-1beta, and COX1.

### 3.3. Hyperuricemia Modifies the Gene Expression Pattern and Interleukin Synthesis by Tendon Cells in Response to PRP Treatment

#### 3.3.1. Hyperuricemic PRP and Inflammation

**Figure 2:**
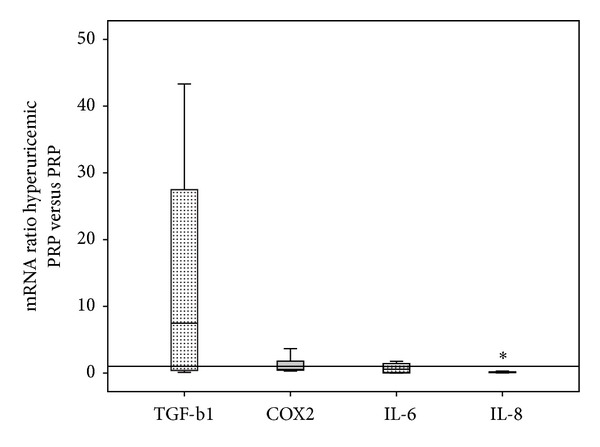
Boxplots of modulators of inflammation. Boxes illustrate the relative mRNA expression of modulators of inflammation (TGF-b1, COX2, IL-6, and IL-8); the band inside the box is the median. mRNA folds of hyperuricemic PRP treated cells are calculated relative to PRP treated cells. IL-8 expression is significantly reduced in cells treated with hyperuricemic PRP. *P < 0.05.

**Figure 3:**
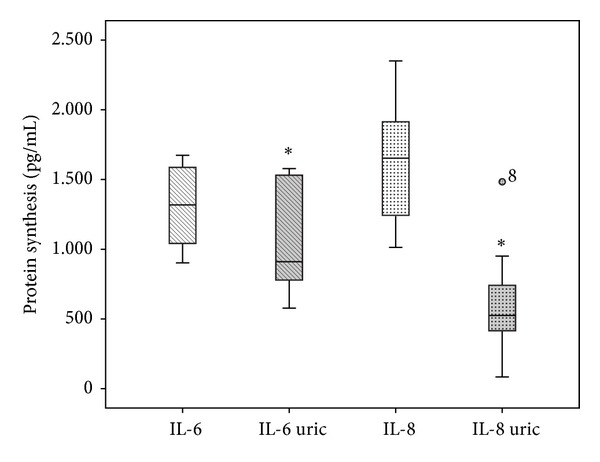
Synthesis of IL-6 and IL-8 proteins. The concentration of IL-6 and IL-8 is reduced in tendon cells treated with hyperuricemic PRP compared to cells treated with PRP. Data are compared using the Wilcoxon signed-rank test for matched samples. *P < 0.05.

The levels of IL-1beta and COX1 were not detectable (Ct > 32) when tendon cells were treated with PRP or hyperuricemic PRP. COX2 was significantly stimulated by hyperuricemia in T1 but not in T2. There was evidence of expression and synthesis of two pleiotropic interleukins, IL-8 (CXCL8) and IL-6 (CXCL6), in the conditioned media. Constitutive synthesis of interleukins was IL-6, 416 pg/mL (range 328–503) and IL-8 166 pg/mL (range 123–196). PRP induced a 3-fold increase over constitutive values for IL-6 and 9.6-fold for IL-8. Notwithstanding, hyperuricemic PRP induced a 2-fold increase of IL-6 and 3-fold increase of IL-8 over constitutive values. Remarkably, the expression and synthesis of IL-6 and IL-8 induced by PRP are decreased significantly in the presence of hyperuricemia (Figures 2 and 3).

These findings corroborate changes in the inflammatory response to PRP induced by hyperuricemia.

There was evidence of a statistically significant positive association between COX2 and IL-6 (*R* = 0.8330, *P* = 0.010) and TGF-b1 (*R* = 0.81, *P* = 0.015). There was evidence of a statistically significant negative association between IL-6 and scleraxis (*R* = −0.711, *P* = 0.048) and aggrecan (*R* = −0.726, *P* = 0.041). There was evidence of a statistically significant positive association between the expression of COLA1 and aggrecan (*R* = 0.881, *P* = 0.004) and scleraxis (*R* = 0.714, *P* = 0.047). Additionally, there was evidence of a statistically significant positive association between the expression of COMP and the expression of HAS2 (*R* = 0.826, *P* = 0.011).

Taken together, these results could indicate coregulation of some proteins and can be used to infer that a greater inflammatory cell response to the molecular environment is associated with cell dedifferentiation and a decreased synthesis of aggrecan.

#### 3.3.2. Changes in the Pattern of Expression of the Genes Codifying Extracellular Matrix Proteins

**Figure 4:**
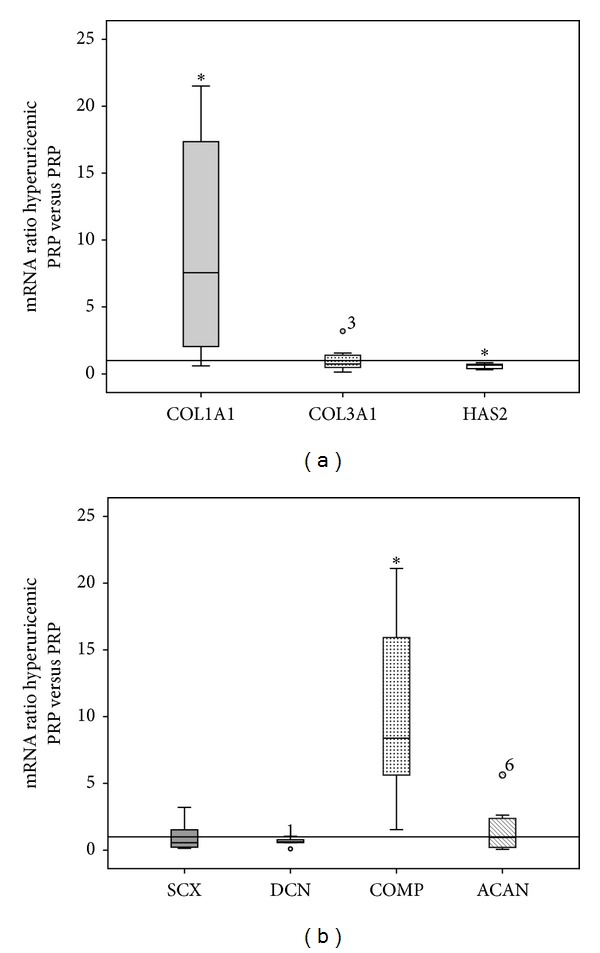
Relative expression of (a) fibrillar extracellular matrix proteins and (b) nonfibrillar extracellular matrix proteins in tendon cells treated with hyperuricemic PRP compared with cells treated with PRP. *P < 0.05.

In hyperuricemic PRP conditions, the expression of type 1 collagen was significantly increased (*P* = 0.036) (Figure 4(a)). Similar to type 1 collagen, COMP expression was significantly increased by hyperuricemia, *P* = 0.012. The expression of COL3A1, the gene codifying type 3 collagen, is not altered by hyperuricemia, *P* = 0.674. In addition, the expression of decorin did not change (*P* = 0.080) (Figure 4(b)). There was evidence of a statistically significant positive association between TGF-b1 and type 1 collagen (*R* = 0.811, *P* = 0.015) and TGF-b1 and aggrecan (*R* = 0.761, *P* = 0.028).

The expression of HAS2, the enzyme involved in hyaluronan synthesis, was significantly reduced in hyperuricemic PRP compared with PRP (*P* = 0.012).

## 4. Discussion

We explored whether tendon cells can sense hyperuricemia in their biological milieu and whether hyperuricemic PRP can incite tendon cells to switch to an inflammatory phenotype. We found that PRP induces a modest inflammatory molecular response in tendon cells compared to constitutive values and that hyperuricemia can mitigate this reaction. Furthermore, we report that hyperuricemia modifies the expression pattern of extracellular matrix proteins induced by PRP treatment.

One possible way of investigating whether hyperuricemia may affect inflammation and tendon metabolism is to expose primary tendon cells to hyperuricemic PRP* in vitro*.

To achieve the greatest approximation to the* in vivo* cell we have used cells up to passage 3 [20, 21].

These cells exposed to PRP for 96 hours could be an acceptable representation of the* in situ* tenocytes treated with PRP. Most commonly, tendon cells are used between passages 3 and 5, showing a more homogeneous behaviour, but being less representative of* in vivo* conditions. Actually, we have found some heterogeneity between cell donors, which can be attributed to the early passage (passages 1–3).

One of the central observations in this study is that PRP induces modest inflammation, evidenced by the production of IL-6, IL-8, COL1A1, and COX2. Analogous to our* in vitro* model, in painful Achilles tendinopathy, the expression of COL1A1, COX2, and IL-6 increased compared with normal tendon [16]. This could be attributed to the set of circumstances underlying the failed healing that occurs in tendinopathy and to the subsequent positive feedback reaction. In fact, an inflammatory molecular response expressed by local cells is crucial for tissue healing; PRP can reproduce this modest inflammatory response driving tendon cells to produce IL-6 and IL-8. The latter is known not only because of its ability to attract neutrophils but also for its proangiogenic properties. Actually, IL-8 could act in synergy with angiogenic factors such as HGF and VEGF produced by tendon cell in response to PRP [22, 23]. Angiogenesis and inflammation are closely linked in the early phases of tissue repair. Interestingly, hyperuricemia reduced significantly the expression and synthesis of IL-8. Whether the reduction in IL-8 in the context of hyperuricemia has relevant consequences derived from reduced neutrophil infiltration and diminished angiogenesis* in vivo* is still unexplored.

The synthesis of IL-6 is also reduced by hyperuricemia. IL-6 may play a crucial role in tendon healing as tendon healing was significantly reduced in IL-6 knockout mice [22]. Moreover, IL-6 plays a role as mediator of the anti-inflammatory effects of exercise [15] and is increased along with COL1A1 expression in painful tendons [16]. Moreover, tendon is a highly mechanosensitive tissue, and both IL-6 and TGF-beta1 have been involved in transforming mechanical loading into collagen synthesis after exercise [24]. Corroborating these findings, our experiments showed coregulation between these molecules embodied by evidence of statistically significant associations in gene expression.

How cells sense uric acid is not clear. In particular, our experiments show that tendon cells sense uric acid but whether it could occur via TLR2 as in chondrocytes is unexplored [25]. Actually, tenocytes express the main receptors involved in sterile inflammation, TLR2 and TLR4, [26], but it is not clear in what circumstances these receptors are functional. Uric acid can also enter the cell via specific transporters where it can modulate inflammatory and oxidative events.

Priming TLR2 and TLR4 with other molecules such as SAA induced TLR-dependent production of IL-6 and IL-8 and facilitated the inflammatory actions of MSU in synoviocytes [13]. Hyperuricemic PRP can induce sterile inflammation if uric acid behaves as an immunological danger signal. While some data about the interactions of immune cells [12], chondrocytes [25], or synovial fibroblasts with MSU crystals are available [13], little is known about the response of tendon cells to hyperuricemic fluids. Recent data show that tendon-like fibroblasts interact with MSU decreasing the expression and deposition of collagens [27]. Corroborating these findings, we found further induction of COX2 expression, but not of IL-1b and COX1, when tendon cells were further exposed to 100 ug of monosodium urate crystals (MSU) for 24 h after 96-hour treatment with hyperuricemic PRP (data not shown). In these conditions, the expression of IL-6 and IL-8 dropped below detection limits.

Our results corroborate the idea that only crystallised uric acid induces inflammation. Indeed, in our experimental conditions, hyperuricemic PRP further enhanced the synthesis of type I collagen and reduced the synthesis of IL-6 and IL-8. This reduction may hinder the regenerative effects of PRP, assuming that they are linked to angiogenesis and inflammation.

The present results set the rationale for performing future* in vivo* research aiming to assess whether the depletion of IL-6 and IL-8 hinders the regenerative effects of PRP in tendon lesions. As further data were collected about the angiogenic or parainflammatory responses induced by PRP, we found that hyperuricemia is a minor stressor for tendon cells [28].

Our study has several limitations and from these data it is difficult to reach conclusions to be extrapolated to* in vivo* conditions. Some uncertainties can be unveiled by coculturing tenocytes with monocytes/macrophages, as they synthesise major inflammatory triggers such as IL-1beta with paracrine actions on tenocytes. Also, whether the response of healthy and tendinopathic cells to a challenge with hyperuricemic PRP may differ warrants additional studies. Moreover, patients with hyperuricemia may have some systemic comorbidities including diabetes or metabolic syndrome, and their PRP will reflect these alterations.

In conclusion, we show that hyperuricemic PRP may exert a positive effect on tendons by increasing the production of type 1 collagen and COMP, and at the same time decreasing the production of IL-6 and IL-8. Thus, a priori, patients with hyperuricemia shall not be excluded from PRP treatment. However, not only local tenocytes but also infiltrated innate immune cells respond to PRP cues. Therefore, depending on the immunological microenvironment and the reciprocal interactions, local cells can acquire distinct functional properties.

## Figures and Tables

**Table 1:** Real-time PCR primers used in this study.

Gene	Forward primer (5′→3′)	Reverse primer (5′→3′)	T (°C)
SCX	CAGCCCAAACAGATCTGCACCTT	CTGTCTTTCTGTCGCGGTCCTT	58
DCN	GGTGGGCTGGCAGAGCATAAGT	TGTCCAGGTGGGCAGAAGTCA	58
TNMD	GAAGCGGAAATGGCACTGATGA	TGAAGACCCACGAAGTAGATGCCA	60
COMP	CCGACAGCAACGTGGTCTT	CAGGTTGGCCCAGATGATG	55
ACAN	ACAGCTGGGGACATTAGTGG	GTGGAATGCAGAGGTGGTTT	55
SOX9	AGCGAACGCACATCAAGAC	GCTGTAGTGTGGGAGGTTGAA	55
COL1A1	GGCAACAGCCGCTTCACCTAC	GCGGGAGGACTTGGTGGTTTT	58
COL3A1	CACGGAAACACTGGTGGACAGATT	ATGCCAGCTGCACATCAAGGAC	58
COL2A1	AACCAGATTGAGAGCATCCG	AACGTTTGCTGGATTGGGGT	55
HAS2	GTCCCG GTGAGACAGATGAG	ATGAGGCTGGGTCAAGCATAG	58
IL-1b	TCCAAGGGGACAGGATATGGAGCA	AGGCCCAAGGCCACAGGTATTT	58
IL-6	GAGGCACTGGCAGAAAACAACC	CCTCAAACTCCAAAAGACCAGTGATG	58
IL-8	CTGTCTGGACCCCAAGGAAAACT	GCAACCCTACAACAGACCCACAC	57
COX1	GGTTTGGCATGAAACCCTACACCT	CCTCCAACTCTGCTGCCATCT	58
COX2	AACTGCGCCTTTTCAAGGATGG	TGCTCAGGGACTTGAGGAGGGT	58
TGF-beta1	GAGGTCACCCGCGTGCTAATG	CACGGGTTCAGGTACCGCTTCT	58
GAPDH	GCATTGCCCTCAACGACCACT	CCATGAGGTCCACCACCCTGT	58
TBP	TGCACAGGAGCCAAGAGTGAA	CACATCACAGCTCCCCACCA	58

Scleraxis (SCX), decorin (DCN), tenomodulin (TNMD), cartilage oligomeric protein (COMP), aggrecan (ACAN), SRY (sex determining region Y)-box 9 (SOX9), collagen type I alpha 1 (COL1A1), collagen type 3 alpha 1 (COL3A1), collagen type II alpha 1 (COL2A1), hyaluronan synthase 2 (HAS2), interleukin 1, beta (IL-1b), interleukin-6 (IL-6), interleukin-8 (IL-8), cytochrome c oxidase 1 (COX1), cytochrome c oxidase 2 (COX2), glyceraldehyde 3-phosphate dehydrogenase (GAPDH), and TATA box binding protein (TBP).

**Table 2:** Relative gene expression normalised to the mean of GAPDH and TBP (2^−ΔCt^) given as medians and 25–75 percentiles.

Cell donor	SCX	HAS2	COLA1	COLA3	COMP	DCN	IL6	COX2	IL8	ACAN	TGF-b1
T1	**0.073** (0.057–0.113)	**1.308** (0.816–1.610)	**158.67** (136.07–166.94)	**7.052** (3.252–11.519)	**0.025** (0.020–0.030)	**2.256** (1.670–2.789)	**0.166** (0.149–0.172)	**0.088** (0.037–0.112)	**0.045** (0.039–0.057)	**0.0202** (0.0124–0.0342)	**0.6676** (0.4728–1.3547)

T2	**0.049** (0.035–0.068)	**1.710** (1.493–2.232)	**74.792** (40.05–113.63)	**3.558** (2.831–5.468)	**0.025** (0.023–0.038)	**1.697** (1.444–2.216)	**0.805** (0.481–1.254)	**0.149** (0.117–0.227)	**2.058** (1.885–2.480)	**0.007** (0.006–0.008)	**0.590** (0.504–0.676)

T3	**0.005** (0.003–0.136)	**2.055** (1.909–2.974)	**137.16** (104.5–154.5)	**1.517** (1.174–1.60)	**2.003** (1.91–5.17)	**0. 489** (0.419–0.854)	**0.052** (0.032–0.076)	**0.048** (0.045–0.343)	**0.02** (0.016–0.159)	**0.568** (0.172–0.812)	**0.828** (0.616–10.98)

Relative expression levels of tendon cells from three donors (T1, T2, and T3) treated with allogeneic PRP (six donors) for 96 hours, normalised to the mean of GAPDH and TBP (2^−ΔCt^) given as medians and 25–75 percentiles.

Scleraxis (SCX), decorin (DCN), tenomodulin (TNMD), cartilage oligomeric protein (COMP), aggrecan (ACAN), SRY (sex determining region Y)-box 9 (SOX9), collagen type I alpha 1 (COL1A1), collagen type 3 alpha 1 (COL3A1), collagen type II alpha 1 (COL2A1), hyaluronan synthase 2 (HAS2), interleukin-1, beta (IL-1b), interleukin-6 (IL-6), interleukin-8 (IL-8), cytochrome c oxidase 1 (COX1), cytochrome c oxidase 2 (COX2), glyceraldehyde 3-phosphate dehydrogenase (GAPDH), and TATA box binding protein (TBP).
